# Using qualitative research to develop an elaboration of the TIDieR checklist for interventions to enhance vaccination communication: short report

**DOI:** 10.1186/s12961-022-00833-2

**Published:** 2022-03-19

**Authors:** Claire Glenton, Benedicte Carlsen, Brita Askeland Winje, Renske Eilers, Manuela Dominique Wennekes, Tammy C. Hoffmann, Simon Lewin, The VITAL Consortium

**Affiliations:** 1 Norwegian Institute of Public Health, Oslo, Norway; 2 TRS National Resource Centre for Rare Disorders, Sunnaas Rehabilitation Hospital, Nesoddtangen, Norway; 3 Department of Health Promotion and Development, University of Bergen, Bergen, Norway; 4 Faculty of Health Sciences, Oslo Metropolitan University, Oslo, Norway; 5 Centre for Infectious Disease Control, National Institute for Public Health and the Environment (RIVM), Bilthoven, Netherlands; 6 Athena Institute, Free University, Amsterdam, Netherlands; 7 Institute of Evidence-Based Healthcare, Bond University, Gold Coast, Australia; 8 Health Systems Research Unit, South African Medical Research Council, Cape Town, South Africa

**Keywords:** Qualitative evidence synthesis, Qualitative research, Trial reporting, TIDieR checklist, Vaccination communication, Vaccines, Standards, Randomized controlled trials, Methods

## Abstract

**Background:**

The COVID-19 pandemic has led to an increased interest in communication with the public regarding vaccination. Our recent Cochrane qualitative evidence synthesis points to several factors that could influence the implementation and success of healthcare worker communication with older adults about vaccination. However, it is often difficult to assess whether factors identified as potentially important in qualitative studies have been considered in randomized trials because of poor trial reporting. We therefore decided to use our qualitative evidence synthesis findings to encourage better reporting of vaccination communication interventions in trials by developing an elaboration of the TIDieR (Template for Intervention Description and Replication) checklist for intervention reporting.

**Methods:**

We examined the findings from our Cochrane qualitative evidence synthesis on healthcare workers’ perceptions of and experiences with communicating about vaccination with adults over the age of 50 years. We identified factors that could influence the implementation and uptake, and thereby the effectiveness, of vaccination communication interventions. We then drafted a list of the information elements we would need from trial reports to assess whether these factors had been considered in the development of the interventions evaluated in these trials. Finally, we compared our list of information elements to the TIDieR checklist items. We were able to align all of our information elements with the TIDieR items. However, for several of the TIDieR items, we developed a more detailed description to ensure that relevant information would be captured sufficiently in trial reports.

**Results:**

We developed elaborations for the following TIDieR items: “Why” (item 2), “What—materials” (item 3), “Who provided” (item 5), “How” (item 6), “Where” (item 7) and “Tailoring” (item 9).

**Conclusions:**

Both qualitative research and trials of intervention effectiveness are critical to furthering our understanding of what works, where, for whom and through which mechanisms. However, a key ingredient for developing this understanding is adequate reporting of intervention design, content and implementation in randomized trials. We hope that this elaboration of the TIDier checklist will improve reporting of interventions in trials focused on vaccine communication with older adults, and thereby enhance the usability of this research for developing future communication strategies.

## Contributions to the literature

- Qualitative research can identify factors influencing the implementation and uptake, and thereby the effectiveness, of healthcare interventions. But it is often difficult to tell whether these factors have been considered in randomized trials because of poor intervention reporting.
- The Template for Intervention Description and Replication (TIDieR) checklist aims to improve the completeness of intervention reporting. The checklist is flexible enough to apply to most interventions but may not be able to capture all aspects of particularly complex interventions.
- We used a Cochrane qualitative evidence synthesis to develop an elaboration of the TIDieR checklist for reporting interventions to enhance health worker communication with older adults about vaccination.

## Background

The COVID-19 pandemic has led to increased interest in how health agencies, governments and other authorities communicate to the general public about preventive behaviour, including vaccination [1, 2]. An important part of this picture is the communication that takes place between healthcare workers and adults. We recently finished a Cochrane qualitative evidence synthesis that explores healthcare workers’ perceptions of and experiences with communicating about vaccination with adults over the age of 50 years [3]. This review was carried out as part of a European Union-funded project entitled VITAL (Vaccines and InfecTious diseases in the Ageing popuLation) that aims to develop strategies to train and educate healthcare workers about vaccines and vaccination communication for older adults. Our review findings offer potentially valuable lessons for people who are designing healthcare worker communication strategies or evaluating the effects of these strategies.

### Using qualitative research to understand the results of randomized trials

Our qualitative evidence synthesis suggests that few qualitative studies have explored the topic of vaccination communication between healthcare workers and older adults (defined in our review as adults over the age of 50 years). Nevertheless, the qualitative studies we did identify point to several factors that could potentially influence the implementation and success of vaccination communication strategies. These include factors tied to the amount and content of the information, the type of healthcare worker, the nature of the healthcare worker–patient relationship, and healthcare workers’ views of and experiences with the disease and the vaccine, as well as organizational and practical issues, all of which appeared to influence communication between healthcare workers and older adults. Healthcare workers’ perceptions of the aim of vaccination communication—for instance, whether they saw it as a method for achieving “compliance” or supporting informed choice—also appeared to influence communication.

After we had completed our synthesis, our original plan was to assess whether these factors were considered in the communication strategies that were being assessed in randomized trials of vaccination communication interventions for older adults. Could consideration (or lack of consideration) of these factors offer explanations for why these communication strategies did or did not work in the context of a trial?

At least one systematic review has searched for and summarized trials assessing the effectiveness of interventions to increase vaccine uptake among older adults [4]. While this review does not focus specifically on communication strategies, several of the included trials include communication elements in their intervention design. In addition, a systematic review carried out as part of the VITAL project focuses on the effectiveness of educational and training interventions for healthcare workers communicating with older adults about vaccination [5].

We initially aimed to use a matrix model approach to link the findings from our qualitative evidence synthesis to the trials included in these two reviews. In this approach, previously described by Candy et al. [6], review authors use the findings from a qualitative evidence synthesis to suggest components that interventions dealing with a specific health issue should include. Review authors then plot these components into a matrix to assess whether these components correspond with the components of the interventions evaluated in relevant randomized trials. A matrix model approach was considered useful, as it could suggest a link between the effectiveness of the interventions and the extent to which these interventions contained the components identified as important by participants in the qualitative studies. However, Candy et al. also point out that incomplete reporting in randomized trial reports poses a threat to the validity of this approach, as it depends on the extent and quality of the information provided on each trialled intervention [6]. Candy et al. [6] therefore limited themselves to trials that used at least one paragraph to describe the intervention and that met reporting requirements described in the CONSORT (Consolidated Standards of Reporting Trials) statement’s extension developed specifically for non-pharmacological treatments [7].

Inspired by this and other studies [8], we have previously used a matrix model approach in qualitative evidence syntheses. We first identified the factors identified in these syntheses as potentially important to interventions for the health issue considered by the synthesis. We then assessed whether these factors corresponded with the components of interventions evaluated in trials included in related effectiveness reviews [9–11]. However, our attempts had mixed success because of the poor reporting of the interventions in the trial reports. We were therefore uncertain whether the factors we identified as potentially important in the qualitative research were not considered by triallists when designing the intervention, or were considered (and perhaps even implemented) but simply not reported. We therefore decided to set aside the planned matrix model in our qualitative evidence synthesis on vaccination communication. Instead, we opted to use our qualitative evidence synthesis findings to encourage better reporting of vaccination communication interventions in effectiveness trials by developing an elaboration of the Template for Intervention Description and Replication (TIDieR) checklist for intervention reporting [12].

### Using qualitative research to develop a reporting checklist for vaccination communication trials

Item 5 of the CONSORT 2010 statement asks the authors of trial reports to describe the intervention “with sufficient details to allow replication, including how and when they were actually administered” [13]. The CONSORT statement has been endorsed by many healthcare journals. However, item 5 lacks detailed guidance. The TIDieR checklist was therefore developed by an international group of experts and stakeholders to further improve the completeness of intervention reporting [12].

The TIDieR checklist encourages triallists to report 12 items to describe the intervention: brief name, why, what (materials), what (procedure), who provided, how, where, when and how much, tailoring, modifications, how well (planned), and how well (actual) [12]. These items are flexible enough to apply to most interventions. However, the checklist authors acknowledge that for some particularly complex interventions [14], the checklist may not be able to capture the full complexity of these interventions [12]. The need for versions of the checklist that are specific to particular topics has therefore been suggested [15].

In order to consider whether the factors identified in our qualitative evidence synthesis are addressed in trials of vaccination communication interventions, we require relatively detailed information regarding these interventions. While this information is broadly covered by the TIDieR checklist, some of the items need further elaboration. Developing an elaboration of the TIDieR checklist that is tailored to our particular topic may therefore be helpful in improving intervention reporting in the area of vaccination communication for older adults.

## Methods

In step 1, we used an approach that we had developed previously [16]. Here, members of the review team assessed each of the findings from our qualitative evidence synthesis on healthcare workers’ perceptions of and experiences with communicating about vaccination with adults over the age of 50 years [3]. This assessment aimed to identify factors that might influence the implementation and uptake, and thereby the effectiveness, of vaccination communication interventions. For instance, one of our findings (see Table 1) described the role of trust between the healthcare worker and the older adult and how this was linked to the length of their relationship. Based on this and other findings, we identified the healthcare worker–patient relationship as one factor that could potentially influence the implementation, uptake and effectiveness of vaccination communication interventions. We included findings that were assessed as low-, moderate- or high-confidence using the GRADE-CERQual [Grading of Recommendations Assessment, Development and Evaluation–Confidence in the Evidence from Reviews of Qualitative research] approach [17].

**Table 1:** Moving from review finding to TiDieR elaboration—example

Review finding	Step 1: What factors does the review finding describe that might influence the implementation and uptake of the intervention?	Step 2: What information would we need from the trial reports to assess whether these factors had been considered in the development of the intervention?	Step 3: Which, if any, TIDieR checklist items does this information relate to?	Step 4: How could these TiDieR items be elaborated to ensure that this information is captured?
Finding 4. Healthcare workers in community-based and primary care settings described how older adults often followed their vaccine recommendations. Healthcare workers believed that this influence was linked to trust, which in turn was linked to long-lasting relationships and sometimes also to shared cultural or language backgrounds (low-confidence finding)	1. The nature of the patient–healthcare worker relationship 2. The extent to which the healthcare worker and patient speak a shared language	3. What is the nature of the relationship between the healthcare worker and the older adult? Do they already have an established relationship (for instance, are they the older adult’s family doctor or nursing home staff), or is it likely that the older adult will be meeting them for the first time (for instance, during a hospital appointment)? 4. Have any routines been put in place to facilitate communication with older adults who do not speak the majority language?	TiDieR item 5. Who provided: For each category of intervention provider (for example, psychologist, nursing assistant), describe their expertise, background and any specific training given TiDieR item 9. Tailoring: If the intervention was planned to be personalized, titrated or adapted, then describe what, why, when and how	TiDieR item 5. Who provided: Describe the relationship between the healthcare worker and the older adult. Do they already have an established relationship (for instance, are they the older adult's family doctor or nursing home staff), or is it likely that the older adult will be meeting them for the first time (for instance, during a hospital appointment)? TiDieR item 9. Tailoring: Describe any routines that have been put in place to facilitate communication with older adults who do not speak the majority language

In step 2, we discussed these factors within the review team. Based on this discussion, we then drafted a list of the information elements we would need from the trial reports to assess whether the factors we had identified as potentially important had been considered in the development of the interventions. For instance, in order for us to assess the nature of the healthcare worker–patient relationship, we determined that it would be helpful to have information about the extent to which the healthcare workers in the trials already had an established relationship with the older adult (for example, whether they were the older adult’s family doctor or nursing home staff), or whether the older adult would be meeting them for the first time (for instance, during a hospital appointment).

In step 3, we compared our list of information elements to the TIDieR checklist items. We were able to align all of our information elements with the items in TIDieR, and for some of these items, we made no further elaboration because the findings from our review did not suggest that this was needed. However, for several of the TIDieR items, our information elements provided useful elaborations with regard to adult vaccination communication interventions. In step 4, for these items, we therefore developed a more detailed description to ensure that the information needed would be captured sufficiently in trial reports and other intervention descriptions. We published an earlier version of these elaborations in the Cochrane Review [3], but these have since gone through further iterations.

## Results

The TIDieR items where we added elaboration were item 2 (“Why”), item 3 (“What—materials”), item 5 (“Who provided”), item 6 (“How”), item 7 (“Where”) and item 9 (“Tailoring”). Our elaboration of these TIDieR checklist items is shown in Table 2. These elaborations should be used in conjunction with the explanations provided for each item in the published TIDieR checklist [12].

**Table 2:** Elaboration of the TIDieR checklist when reporting interventions to enhance health worker communication with older adults about vaccination

TIDieR checklist items [12]	Elaboration of TIDieR checklist items when reporting interventions to enhance vaccination communication with older adults^a^
Item 1. Brief name: Provide the name or a phrase that describes the intervention	(No elaboration made)^b^
Item 2. Why: Describe any rationale, theory or goal of the elements essential to the intervention	Define and make explicit the overall aim of the intervention. Does the intervention primarily aim to increase vaccination uptake or does it aim to support the individual’s informed choice, for instance by giving them access to evidence-based, unbiased information and the opportunity for shared decision-making?
Item 3. What (materials): Describe any physical or informational materials used in the intervention, including those provided to participants or used in intervention delivery or in training of intervention providers. Provide information on where the materials can be accessed (for example, online appendix, URL)	Describe the content of any informational material provided to the healthcare worker: Does it include unbiased, evidence-based and up-to-date information about the vaccine’s effectiveness and side-effects? Does it include unbiased, evidence-based and up-to-date information about the severity of the disease in question and its prevalence in your setting? Does it cover the questions, fears and concerns that older adults commonly have in your setting?
Item 4. What (procedures): Describe each of the procedures, activities and/or processes used in the intervention, including any enabling or support activities	(No elaboration made)^b^
Item 5. Who provided: For each category of intervention provider (for example, psychologist, nursing assistant), describe their expertise, background and any specific training given	Describe the relationship between the healthcare worker and the older adult. Did they already have an established relationship (for instance, were they the older adult’s family doctor or nursing home staff), or is it likely that the older adult was meeting them for the first time (for instance, during a hospital appointment)? Where possible and if they are also a vaccine target group, describe the proportion of healthcare workers who had received the vaccine themselves Describe the content of any training given to the healthcare worker: Did it discuss their responsibility for vaccination communication in relation to other healthcare workers or other parts of the health services? Had relevant stakeholders, such as professional bodies, been involved in the content and delivery of the training? Did it emphasize the value of providing unbiased, evidence-based information about the disease and the vaccine? Did it include a clarification of the aim of the communication; and a discussion of their role and the role of the older adults in vaccination communication and decision-making? Did it reinforce the message that healthcare workers should avoid introducing their own criteria for determining who should and should not receive vaccines?
Item 6. How: Describe the modes of delivery (such as face to face or by some other mechanism, such as internet or telephone) of the intervention and whether it was provided individually or in a group	Describe how the intervention was integrated into the healthcare worker’s routine practice, including: Whether the healthcare worker was expected to raise the issue of vaccination or whether this was usually left to the older adult Whether the healthcare worker had sufficient time to deliver the intervention and how this was determined Describe how healthcare workers accessed relevant patient data, including information about the person’s vaccine history or underlying health conditions
Item 7. Where: Describe the type(s) of location(s) where the intervention occurred, including any necessary infrastructure or relevant features	Describe the extent to which the intervention was delivered to older adults opportunistically (for instance, when attending appointments about other healthcare issues) or at designated time points or events (for instance, during vaccination days)
Item 8. When and how much: Describe the number of times the intervention was delivered and over what period of time including the number of sessions, their schedule and their duration, intensity or dose	(No elaboration made)^b^
Item 9. Tailoring: If the intervention was planned to be personalized, titrated or adapted, then describe what, why, when and how	Describe the content of any informational material that the healthcare worker was expected to share with the older adult and whether the healthcare worker could easily tailor this information to the needs and preferences of the individual older adult Describe any routines that were put in place to address communication issues with older adults who did not have the capacity to make their own decisions Describe any routines that were put in place to facilitate communication with older adults who do not speak the majority language
Item 10. Modifications: If the intervention was modified during the course of the study, describe the changes (what, why, when and how)	(No elaboration made)^b^
Item 11. How well (planned): If intervention adherence or fidelity was assessed, describe how and by whom, and if any strategies were used to maintain or improve fidelity, describe them	(No elaboration made)^b^
Item 12: How well (actual): If intervention adherence or fidelity was assessed, describe the extent to which the intervention was delivered as planned	(No elaboration made)^b^

^a^These elaborations should be used in conjunction with the explanations provided for each item in the published TIDieR checklist [12]

^b^For these items, we made no further elaboration because the findings from our review did not suggest that this was needed

## Discussion

We have developed an evidence-informed elaboration of the TIDieR checklist items [12], focusing on the reporting of interventions to enhance communication between older adults and healthcare workers regarding vaccination. This elaboration is based on the findings of a synthesis of qualitative studies exploring communication between older adults and healthcare workers [3]. We believe that this approach is both a useful method for developing such elaborations and a way of helping to ensure that existing qualitative research informs future intervention design in a specific health area. A potential limitation of this work is that our qualitative evidence synthesis identified few qualitative studies that focused on vaccination communication with older adults. In addition, some of the synthesis findings were assessed to be of low confidence, partly because of concerns about methodological limitations and data adequacy [17]. Future well-conducted primary qualitative studies, including qualitative studies conducted alongside randomized trials, may therefore help to enhance the TIDieR elaboration we have developed.

## Conclusions

Both qualitative research and trials of intervention effectiveness are critical to furthering our understanding of what works, where, for whom and through which mechanisms [18–21]. However, a key ingredient for developing this understanding is adequate reporting of intervention design, content and implementation in randomized trials and other comparative evaluations. Adequate intervention reporting is also critical to replicating and scaling up effective interventions and reducing research waste [22–24]. We hope that this elaboration of the TIDier checklist will encourage improved reporting of communication interventions in trials focused on vaccination for older adults, and thereby enhance the usability of this research for developing future vaccination communication strategies. We encourage future triallists to adopt and pilot the elaborated checklist, and we welcome feedback that may help us improve it.

## Data Availability

The data used to develop this elaboration of the TIDieR checklist are all available in a previously reported Cochrane qualitative evidence synthesis [3].

## Abbreviations

VITAL: Vaccines and InfecTious diseases in the Ageing popuLation
CONSORT: Consolidated Standards of Reporting Trials
TIDieR: Template for Intervention Description and Replication checklist
